# Shaking Table Test of a Transfer-Purge Chamber in Nuclear Island Structure

**DOI:** 10.3390/ma15030766

**Published:** 2022-01-20

**Authors:** Xuchen Liu, Xiaojun Li, Xiaohui Wang, Ning Wang, Zaixian Li

**Affiliations:** 1 Beijing Key Laboratory of Earthquake Engineering and Structural Retrofit, Beijing University of Technology, Beijing 100124, China; liuxuchen1993@163.com (X.L.); lizaixian1992@foxmail.com (Z.L.); 2 Institute of Geophysics, China Earthquake Administration, Beijing 100081, China; wangxiaohui5000@163.com (X.W.); ningwang_cea@163.com (N.W.)

**Keywords:** nuclear power plant, transfer-purge chamber, double steel plates reinforced concrete structure, shaking table test, seismic performance

## Abstract

The transfer-purge chamber is an operation room for nuclear fuel transport and purging in a nuclear power plant, which has a demand for structural reliability and radiation protection. The transfer-purge chamber has features such as large curvature, heavy concrete, long overhang, and irregular cross-sections, and it is constructed of double steel plates reinforced concrete (SC) structure. This study performed shaking table tests for a 1:4.5 scale model of the transfer-purge chamber. Three sets of ground motions were input in the scale model in the horizontal and vertical directions to study its structural reliability and seismic performance. Acceleration response and strain response of the structure were analyzed to evaluate the dynamic characteristics of the transfer-purge chamber under the ground motion. The results show that the transfer-purge chamber has great stiffness and short periods. The periods slightly increase with the rise of intensity of seismic ground motions. Under the excitation of ground motions, the dynamic response of the transfer-purge chamber is slight. No obvious deformation or damage occurred on the transfer-purge chamber, and cracking in concrete or buckling on steel plate did not appear. The transfer-purge chamber has excellent seismic performance, and it is sufficiently safe and reliable from a structural perspective.

## 1. Introduction

Double steel plates reinforced concrete structure (SC structure) is a new composite construction composed of steel plates, inner concrete, and connectors, and it has been applied to nuclear power engineering in the last two decades. In the double steel plates reinforced concrete shear walls (SCSW), concrete mainly bears the axial pressure and prevents out-of-plane instability of the steel plates, yet it gives full play to the bearing capacity, ductility, and energy dissipation capacity of the steel plates [1,2]. There is a confinement effect that a steel plate has on concrete, and it delays the crushing of concrete and buckling of the steel plate. The SCSW has great initial rigidity that effectively reduces the displacement and deformation of the structure under wind loads or earthquake loads. It also has excellent ductility, efficient energy dissipation capacity, and seismic performance [3,4].

Since the 1990s, many researchers have studied the mechanical behavior of SC structures. As shown in existing studies, SC structures have a higher confinement capacity, higher energy dissipation capacity, and stronger seismic performance when compared with traditional reinforced concrete structures (RC structures). To shorten construction periods and reduce construction sites, Akiyama et al. [5] set out to study an SC structure and made a 1/10th scale model of a reactor container, and the result showed that the SC structure had superior ultimate strength and ductile capability. It is one of the earliest studies on SC structures. Usami et al. [6] carried out compressive loading tests for 1/5th scale SC wall specimens, and it was found that the buckling stress of the steel plates can be evaluated using Euler’s equation. It was indicated through shear and bending tests that the buckling and yielding of steel plates have no effect on the overall load-displacement relationship of SC walls and the SC structures have a superior seismic capability compared to RC structures with equal steel ratios [7], which provided a new idea and solution for the seismic resistance of buildings in the future. In addition, the schedule was shortened. Sasaki et al. [8] performed bending shear tests on H-section wall specimens to determine the bending shear characteristics of seismic walls built in an SC structure. Compared with reinforced concrete (RC) composite walls, it was confirmed that the SC structure was superior in yield strength and stiffness by the confinement effect of the steel plates. Takeda et al. [9] tested seven wall-panel specimens under cyclic in-plane pure shear loading to explore the shear behavior of the SC structures. The result showed that the hysteresis loops tend to be larger due to the in-plane shear mechanism of the steel plates, which indicated that SC walls have excellent deformability and in-plane shear resistance. The great shear behavior highlighted the potential of SC structures as resisting shear components, thus researchers focused more on its shear property, deformability, and seismic performance.

Ozaki et al. [10] studied the mechanical performance of SC structures subjected to in-plane shear. It was found that the yield strength linearly increased as the thickness of the steel plates increased, and the cracking strain was affected by axial force. Eom et al. [11] studied the seismic performance of the double steel plates reinforced concrete shear walls (SCSW) through pseudo-static tests under in-plane cyclic loading. The test results indicated that SCSW has a strong energy dissipation capacity, and the failure of the wall specimens was mainly caused by tensile fracture of the welded joints at the wall base and coupling beams or local buckling of the steel plates. Hossain and Wright [12,13,14] conducted a study on the behavior of composite walls in experiment and theory under in-plane shear, and it was found that SCSW can provide higher shear strength and shear stiffness than the summation of the individual contributions from the pair of steel plates and concrete and can provide high shear resistance if adequate boundary connections between steel plates and concrete are ensured.

As shear-resistant members, the out-of-plane shear performance of the SCSW is crucial. Sener et al. [15,16] evaluated the effects of these parameters consisting of wall thickness, shear span-to-depth ratio, and longitudinal reinforcement ratio on the out-of-plane shear strength of SCSW specimens based on the experimental database of China, Japan, South Korea, and the US [17]. It was found that the out-of-plane shear strength decreases with increasing thickness of the section, and the out-of-plane shear strength of the SCSW decreases with increasing shear span-to-thickness ratio, and increasing the longitudinal reinforcement ratio results in higher flexural capacity.

Through pseudo-static tests and relevant numerical simulations, the performance of SCSW was investigated. Zhang and Li [18] found that the spacing of studs is a critical factor that affects the overall strength of the SCSW and delays the local buckling of the steel plates. Zhang et al. [19] studied the effect of shear connectors on the local buckling of steel plates in composite walls. Li’s research results showed that the SCSW has good bearing capacity and lateral stiffness, but its ductility is worse than that of RC shear walls in the failure stage [20,21]. Yang Yue [22] studied the influence of the shear-span ratio, the connection between the steel plate and the concrete, and the thickness of the steel plates on the mechanism, hysteretic characteristics, and the buckling performance of the steel plates of SCSW. Xiong et al. [23,24] found that concrete strength, the thickness of steel plates, axial pressure, and stiffener settings have significant effects on the shear strength of SC structures. At present, China, Japan, South Korea, and the United States have compiled their technical standards for the SC structures of nuclear power plants, and these standards impose strict restrictions on the design of SC structures.

The SC structures have been used in ultra-high-rise buildings, underground engineering, marine engineering, nuclear power plants, and other types of construction. Limited by the scarcity of this kind of engineering, the application of SC structures is rare. As a result, the research on SC structures is not perfect, and there is not much theoretical and practical experience. At present, most of the SC structures are used as local reinforcement members of structures; as a result, the studies of SC structures mainly focus on the ultimate bearing capacity and energy dissipation capacity of the component under various load combinations. There are few studies on the dynamic behavior of SC structures.

The transfer-purge chamber is a crucial part of a nuclear power plant structure developed in China, which was constructed with an SC structure [25]. The transfer-purge chamber comprises two incomplete cylindrical structures that are called the transfer chamber and the purging chamber. There have certain features, like irregular section, large curvature, heavy inner concrete, large overhang, and others, that make it difficult to assess the dynamic characteristics and seismic performance of the transfer-purge chamber based on previous studies. In this study, we designed and constructed a model structure of the transfer-purge chamber with a 1:4.5 scale. Shaking table tests were performed to investigate its dynamic characteristics and seismic performance. The research results are of great significance for revealing the seismic characteristics of SC structures and guiding the structural design of new generation nuclear power plants.

## 2. Design of the Shaking Table Tests

### 2.1. The Prototype

**Figure 1:**
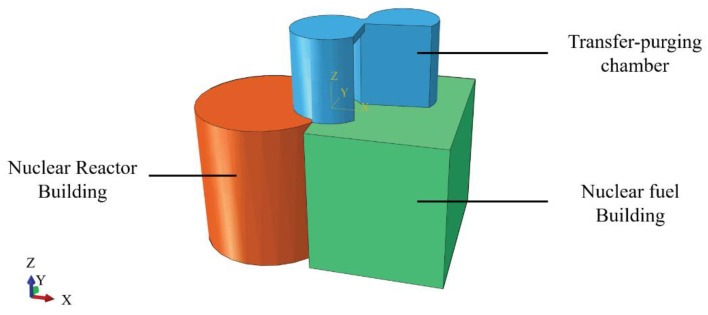
Overall schematic diagram of a nuclear island structure.

The prototype of the shaking table test is the transfer-purge chamber in a nuclear island structure. The transfer-purge chamber is one of the primary facilities in a nuclear island structure and consists of a transfer chamber and a purge chamber. The transfer-purge chamber is located at an elevation of 20.20 m on the top of the nuclear reactor building in a nuclear power plant and connects rigidly to the roof of the nuclear fuel building constructed of an RC structure, as shown in Figure 1. There is saltation of the local stiffness in the connection between SC structure and RC structure, which potentially leads to local damage of the transfer chamber in an earthquake. There is an overhang in the transfer chamber, and its length exceeds 4.8 m. This increases the potential risk of overturning in the transfer-purge chamber. The thickness of the wall of the transfer chamber is 1 m, with an outer radius of 5.1 m and an inner radius of 4.1 m, and the thickness of the wall of the purge chamber is 1 m, with an outer radius of 4.75 m and an inner radius of 3.75 m. The total height of the transfer-purge chamber is 11.2 m, and the thickness of the roof is 1 m. The wall of the transfer-purge chamber was constructed of an SC structure, and the thickness of the inner and outer steel plates is 22 mm. The roof was constructed of an RC structure. The mass of the transfer-purge chamber is about 2460 tons. The transfer-purge chamber has the following characteristics: irregular cross-section, thick wall, long overhang, heavy aggregate concrete, and complex force transmission path. As a channel for nuclear fuel transferring and purging, the transfer-purge chamber has strict technological requirements to prevent radiation leakage. The peak ground acceleration (PGA) of design basis ground motion is 0.3 g for the transfer-purge chamber.

### 2.2. Test Apparatus

**Figure 2:**
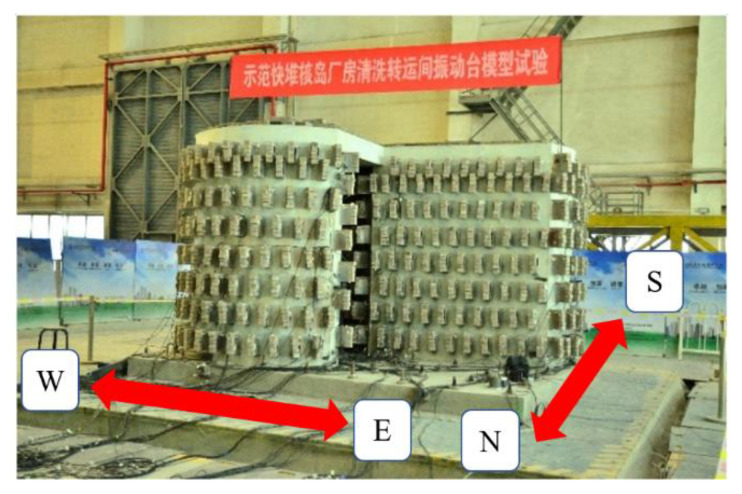
The shaking table tests of the transfer-purge chamber model.

The seismic experiment of the model structure of the transfer-purge chamber was performed on the shaking table in the State Key Laboratory of Building Safety and Environment of China Academy of Building Research. The scale model of the transfer-purge chamber in the tests is shown in Figure 2. The dimension of the shaking table is 6 m × 6 m, and the maximum allowable mass of the shaking table is 60 tons. The operating frequency range is between 0.1 HZ and 50 HZ. The maximum allowable acceleration of the shaking table is 1.5 g in the X-direction, 1.0 g in the Y-direction, and 0.8 g in the Z-direction. The maximum allowable displacement of the shaking table is ±150 mm in the X-direction, ±250 mm in the Y-direction, and ±100 mm in the Z-direction. The maximum overturning moment is 1800 KN·m. The X-direction was along the east-west direction, the Y-direction was along the north-south direction, and the Z-direction was the vertical direction. The shaking table vibrates freely in space with six degrees of freedom.

### 2.3. Similarity Constants

In this study, the without-enough artificial mass model was used to design the model similarity ratio of the transfer-purge chamber [26]. The method is based on the Buckingham π theorem and derives the general expression of the similarity law of the earthquake simulation experiment in the form of the uniform similarity law. The geometric length, peak ground acceleration, and elastic modulus of the materials were selected as the independent basic quantities for similarity design.

To minimize the impact of the scale effect and to make full use of the shaking table, the geometric similarity ratio of the transfer-purge chamber was tentatively determined to be 1/4. Based on a similar relationship, it was calculated that the structural mass of the 1/4 scale model of the transfer-purge chamber exceeded 40 tons. In this case, the peak acceleration of the shaking table cannot exceed 0.6 g. The geometric similarity ratio in the tests was adjusted to 1/4.5. Based on a similar relationship, it was calculated that the structural mass of the 1/4.5 scale model of the transfer-purge chamber was 33T. In this case, the peak acceleration of the shaking table cannot exceed 0.8 g. The geometric similarity ratio of the tests was ultimately selected as 1/4.5 to meet the demand that the peak acceleration of the shaking table exceeds 0.6 g. According to the load-bearing capacity of the shaking table, 1.2 was selected as the similarity ratio of peak ground acceleration in the tests. The fine-grained concrete with a low elastic modulus was used to simulate the heavy concrete in the prototype of the transfer-purge chamber. Based on the elastic modulus of the fine-grained concrete and steel plate, the similarity ratio of the elastic modulus of the model was determined to be 0.32. The rest of the similarity of the model is shown in Table 1.

### 2.4. The Scale Model of the Transfer-Purge Chamber

**Figure 3:**
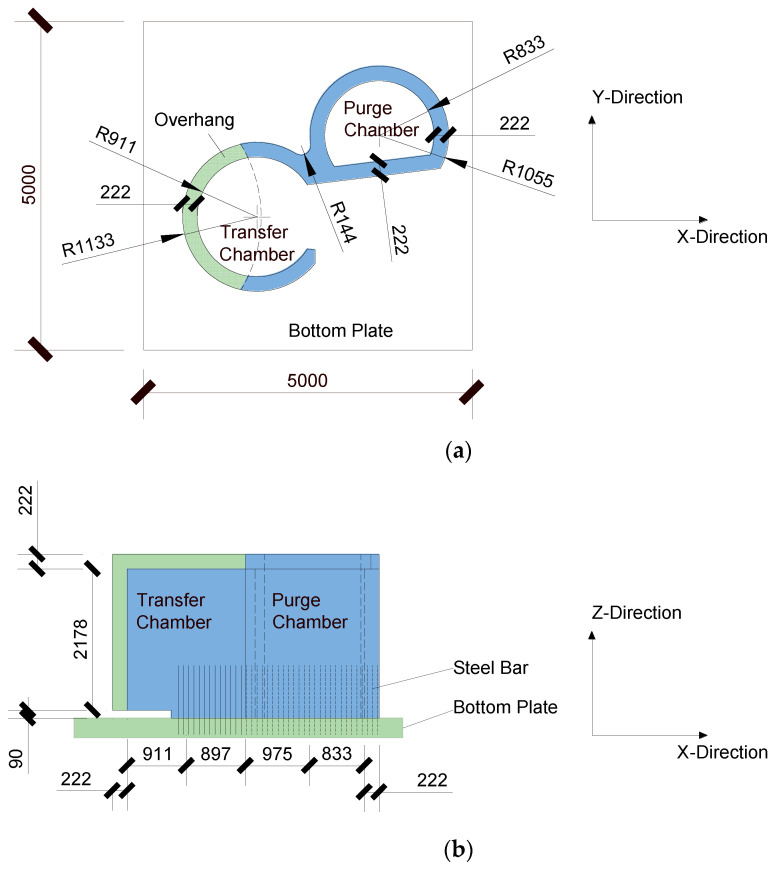
Layout of the transfer-purge chamber model (unit: mm): (**a**) plan view; (**b**) elevation view.

**Figure 4:**
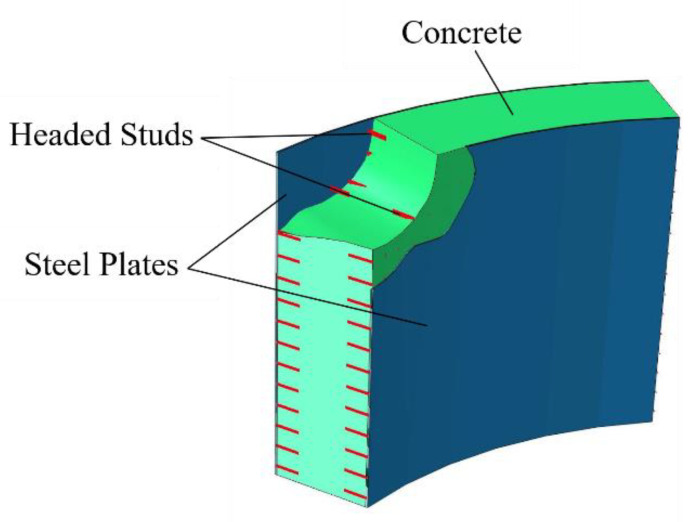
Schematic diagram of double steel plates reinforced concrete wall.

The plan and elevation views of the scale model of the transfer-purge chamber are shown in Figure 3 according to the similarity constants. The outer radius and the inner radius of the transfer chamber model are 1133 mm and 911 mm, respectively, with a wall thickness of 222 mm. The outer radius and the inner radius of the purge chamber model are 1055 mm and 833 mm, respectively, with a wall thickness of 222 mm. The thickness of the roof of the scale model is 222 mm, and the total height is 2480 mm. The wall of the transfer-purge chamber is constructed of an SC structure, and the roof deck is constructed of an RC structure. According to the similarity constants, Q235 steel plates with a thickness of 5 mm and fine-grained concrete with a strength grade of C10 were used in the tests. The full-threaded hex bolt M12 × 80 with a length of 80 mm and a diameter of 6 mm was used to simulate ML15 cheese head studs for arc stud welding with a spacing of 100 mm. The tie bars are HPB300 steel bars with a diameter of 8 mm and a spacing of 100 mm. The scale model was rigidly connected to the shaking table by a 5 m × 5 m reinforced concrete bottom plate with a thickness of 200 mm. The section of the SC wall is shown in Figure 4. In this study, the total weight of the transfer-purge chamber scale model was about 33 tons, of which the structural weight was 18 tons and the counterweight was 15 tons. Considering that the bottom plate of the transfer and cleaning room is relatively thick and rigid, the structural foundation in this paper can be considered as a rigid foundation based on the substructure method.

### 2.5. Instrumentation

**Figure 5:**
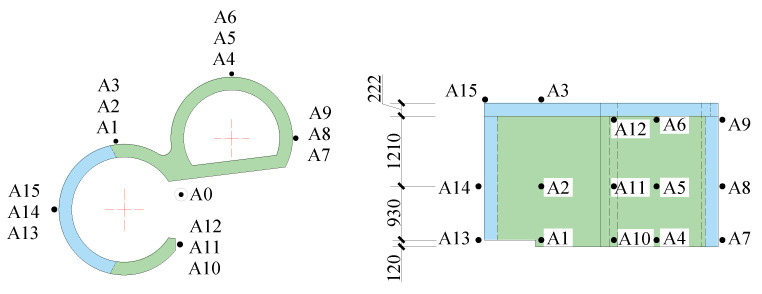
Layout of accelerometers (unit: mm).

**Figure 6:**
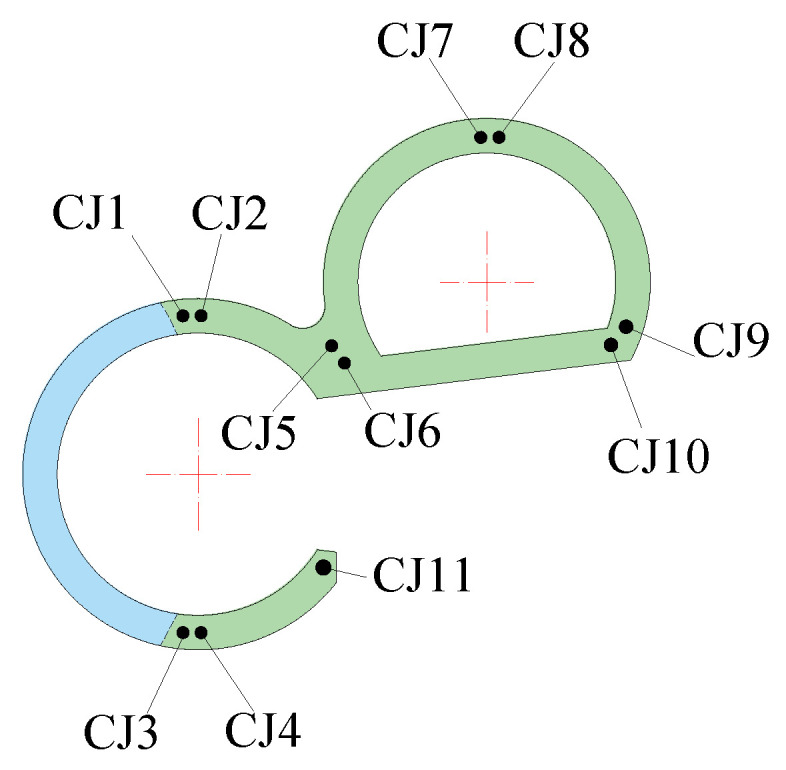
Layout of steel bar strain sensors.

**Figure 7:**
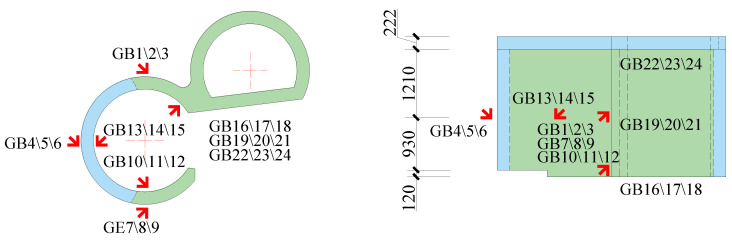
Layout of steel plate strain sensors (unit: mm).

**Figure 8:**
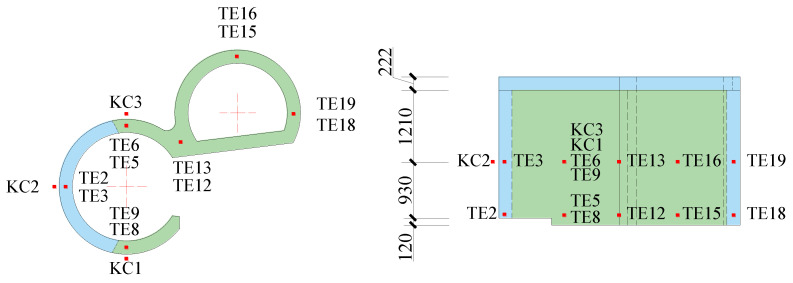
Layout of concrete strain sensors (unit: mm).

Sensors used in these shaking table tests included a piezoelectric accelerometer, an embedded concrete strain gauge, a strain rosette, and a strain gauge. They were used to separately record the acceleration of the model structure, the strain of the anchored steel bars, the strain of the steel plates, and the concrete strain. The sensors contain 16 groups of accelerometers with three directions of two horizontal directions and a vertical direction (X, Y, and Z) per group, 21 concrete strain gauges, 3 strain gauges on the surface of the concrete, 15 steel plate strain gauges, and 11 reinforcement strain gauges. The names of sensors are shown in Table 2. The layouts of the sensors are shown in Figure 5, Figure 6, Figure 7 and Figure 8.

### 2.6. Input Ground Motions and Test Cases

**Figure 9:**
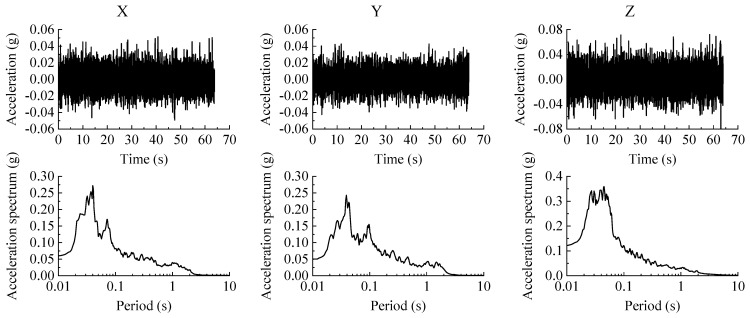
Time history and acceleration response spectra of the white noise motion.

The research purpose of the tests is to study the dynamic characteristics and seismic performance of the transfer-purge chamber. Because the prototype is constructed of an SC structure and the thickness of the wall and the roof deck are both 1 m, the structural stiffness and natural frequencies of the transfer-purge chamber are greater than general civil constructions. It is necessary to focus on the seismic performance of the transfer-purge chamber under the excitation of the ground motions for a short period. The time histories of floor response acceleration obtained from three groups of ground motion (MZQP, MXDB, and YJ) excitation are used as the input ground motions, and the description of the ground motions is shown in Table 3. According to the peak ground acceleration (PGA) of the input ground motions, the test was divided into several steps. White noise was input into the model structure before and after each step to obtain its natural frequencies and structural damping ratios. The time history and acceleration response spectra of the white noise motion are shown in Figure 9.

The PGA was adjusted within the range of 0.24 g to 0.60 g. The duration similarity ratio *S_t_* of the model structure was 0.43, and the duration and acceleration response spectrum of the ground motions were also scaled with *S_t_*. In all cases of the tests, the ground motions were input simultaneously along the X, Y, and Z-directions. The description of the cases in the shaking table tests is shown in Table 4.

**Figure 10:**
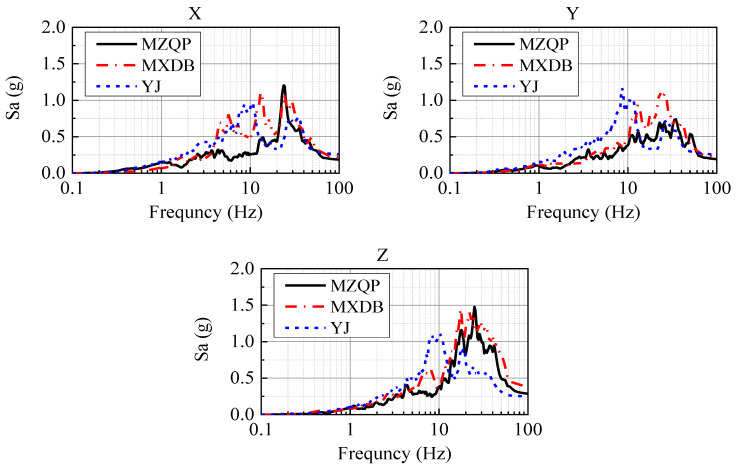
Acceleration response spectra of MZQP, MXDB, and YJ with a PGA of 0.24 g and a damping ration of 4%.

Figure 10 shows the acceleration response spectra of the ground motions MZQP, MXDB, and YJ with a PGA of 0.24 g and a damping ratio of 4%. It was prominent in Figure 9 that the frequency components of the ground motions are rich. In the frequency range of 20~50 Hz, the spectrum of MZQP is close to that of MXDB and greater than that of YJ in the X-direction; the spectrum of MZQP is close to that of YJ and less than that of MXDB in the Y-direction.

The predominant periods and amplitudes of the acceleration response spectra of MZQP, MXDB, and YJ are shown in Table 5.

## 3. Results and Discussion

### 3.1. Seismic Damage

**Figure 11:**
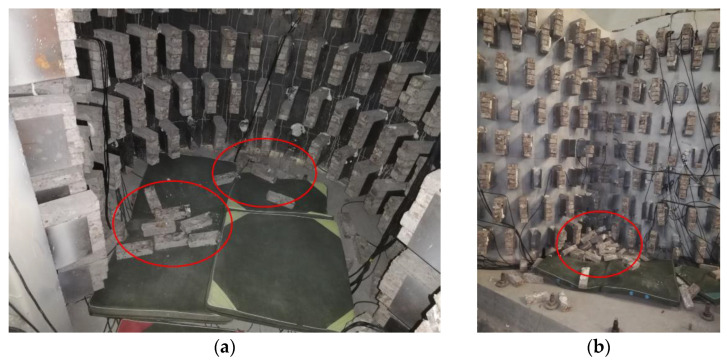
The counterweights fell off: (**a**) inside the transfer chamber; (**b**) external to the transfer-purge chamber.

**Figure 12:**
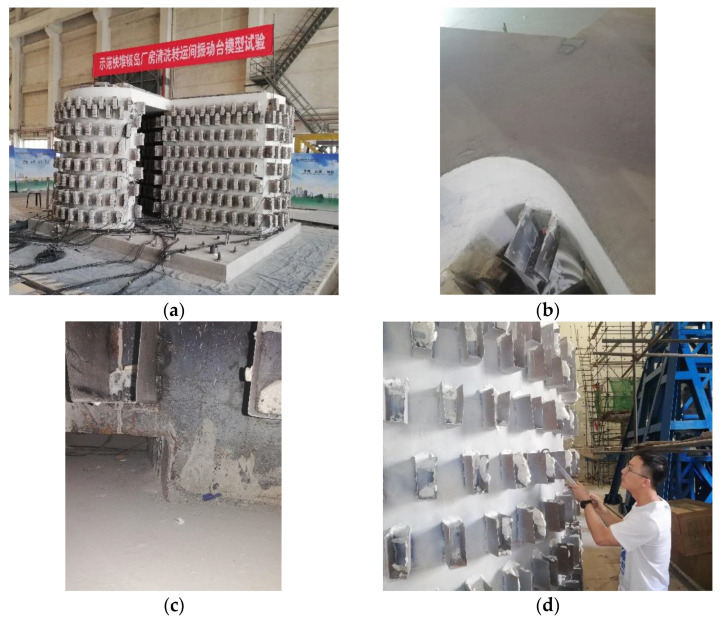
Inspection after the tests: (**a**) no obvious deformation or damage; (**b**) no crack in concrete; (**c**) overhang was intact; (**d**) SC wall worked well.

After the tests, an inspection on the scale model structure of the transfer-purge chamber was performed (Figure 11 and Figure 12). Through the inspection, it was found that the appearance of the scale model structure was intact without obvious deformation or damage. Despite the stress concentration in the structure, the steel plates did not buckle or yield, and the concrete had no crack. We knocked on the steel plates with a metal hammer and judged whether the steel plates and concrete were separated through the knocking sound. It was found that the steel plates and the concrete were closely attached and there was no separation. The steel plates and the concrete worked together well in the scale model of the transfer-purge chamber.

### 3.2. Dynamic Characteristics

The natural frequencies of the scale model structure of the transfer-purge chamber can be obtained through the white noise test of the shaking table, and the damping ratios of the scale model structure can be calculated based on the half-power point method [20]. In engineering, the natural vibration frequency of the undamped system is [27]:

(1) $$f_{n}=\frac{1}{2\pi }\sqrt{\frac{K}{M}}$$

(2) $$f=f_{n}\sqrt{1-\xi^{2}}$$

where *f_n_* is the natural vibration frequency of the undamped system; *f* is the natural vibration frequency of the damped system; *K* is the stiffness of the structure; *M* is the mass of the structure; and *ξ* is the damping ratio of the system. According to Formulas (1) and (2), the structural stiffness of the transfer-purge chamber can be calculated:

(3) $$K=\frac{4\pi^{2}f^{2}M}{1-\xi^{2}}$$

The natural periods and damping ratios of the scale model structure of the transfer-purge chamber are shown in Table 6 and Table 7. The natural period of the transfer-purge chamber scale model is 0.047 s in the X-direction, 0.050 s in the Y-direction, and less than 0.02 s in Z-direction. It can be found that MXDB has great acceleration response spectral amplitudes at the natural period of the scale structure. Similarly, YJ has small acceleration response spectral amplitudes at the natural period of the scale structure in the X and Y-directions.

Before the tests, the natural periods of the transfer-purge chamber scale model were 0.047 s in the X-direction and 0.050 s in the Y-direction. The natural periods of the model structure in the two horizontal directions were close, and the natural period in the X-direction was slightly less than that in the Y-direction. The structural cross-section of the transfer-purge chamber is irregular, the symmetry is poor, and the mass distribution and stiffness in the horizontal direction are unbalanced.

With the increase in PGA, the natural frequencies and stiffness of the transfer-purge chamber scale model structure gradually decreased, and the structural damping ratios gradually increased. The maximum of PGA reached 0.6 g, and the scale model structure was intact. The natural frequency remained 91.1%, and stiffness remained 83% of the initial values in the X-direction. The natural frequency remained 96.9%, and stiffness remained 94% of the initial values in the Y-direction. It was found that the stiffness loss and structural damage of the model structure in the Y-direction were significantly lower than those in the X-direction after the tests. The reduction in stiffness in the X-direction indicates that there may be slight damage to the connection of the transfer-purge chamber and the base. After the tests, the damping ratio of the model structure increased by 34.8% in the X-direction and 21.7% in the Y-direction.

### 3.3. Acceleration Response

**Figure 13:**
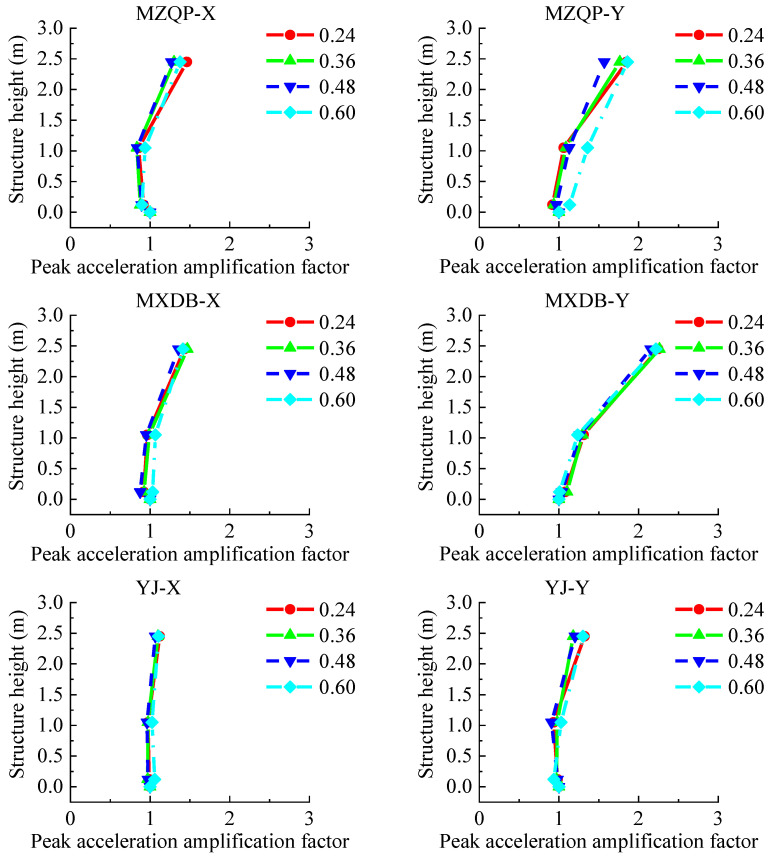
Peak acceleration amplification factors of the model structure along the structure height (7–9 points).

**Figure 14:**
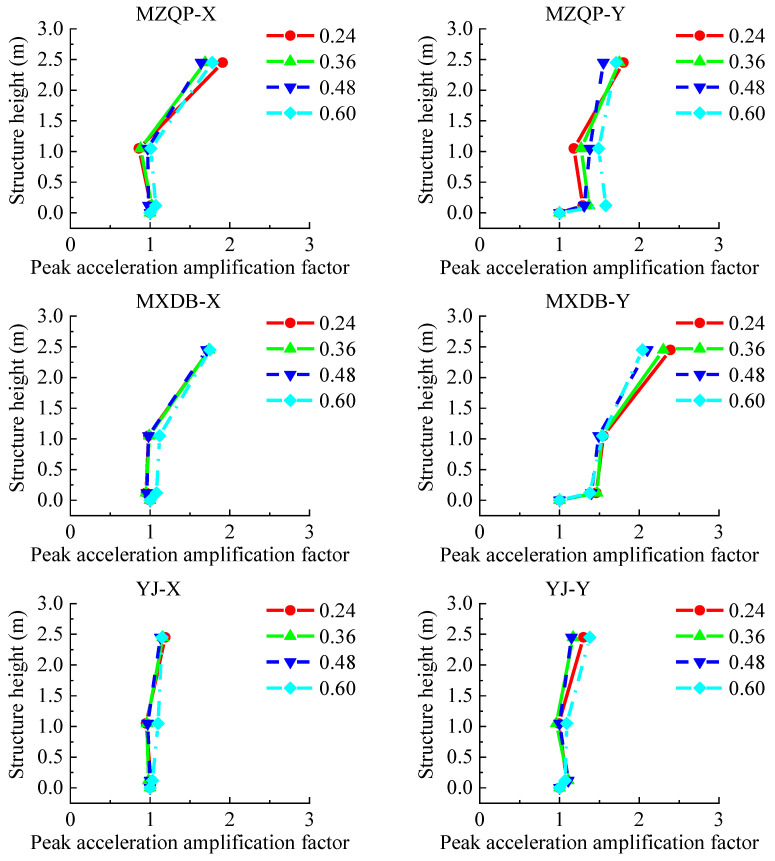
Peak acceleration amplification factors of the model structure along the structure height (13–15 points).

The output peak acceleration at the platform of the shaking table was taken as the reference acceleration to study the acceleration response law of the transfer-purge chamber model structure. The peak acceleration amplification factor was defined as the ratio of the peak acceleration of the model structure to the reference acceleration. The layout of accelerometers is shown in Figure 5. The acceleration amplification factors are shown in Figure 13 and Figure 14.

The PGA in the tests ranged from 0.24 to 0.60 g. As shown in Figure 13 and Figure 14, the peak acceleration amplification factors of the transfer-purge chamber scale model are small and gradually increase along with the structural height. The law of the peak acceleration amplification factors in the structure varies slightly by different ground motions. With the increase in PGA, the peak acceleration amplification factors do not increase. By comparison, it was found that the peak acceleration amplification factors in the Y-direction are significantly less than that in the X-direction. This means that the dynamic response of the model structure in the Y-direction is significantly greater than that in the X-direction. It was found that the peak acceleration amplification factors under MZQP are greater than those under MXDB and YJ in the X-direction, and the peak acceleration amplification factors under MXDB are greater than those under MZQP and YJ in the Y-direction. These results are consistent with the response frequency spectrum characteristics of the ground motions of MZQP, MXDB, and YJ.

### 3.4. Strain Response

#### 3.4.1. Anchored Bars

**Figure 15:**
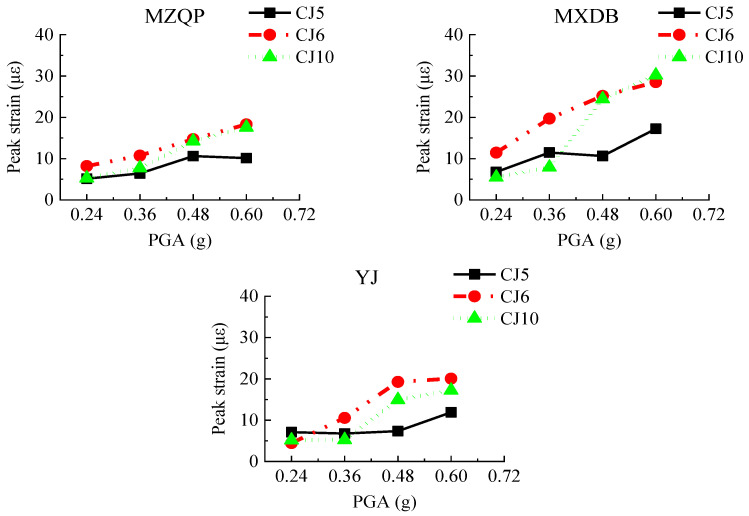
Peak strains of anchored bars.

The transfer-purge chamber structure was rigidly connected to the nuclear fuel building by anchored bars and concreting that held the transfer-purge chamber and the roof deck of the nuclear fuel building together. To evaluate whether the transfer-purge chamber is at risk of overturning and whether the connection is sufficiently strong between the transfer-purge chamber structure and the nuclear fuel building, the strain gauges were arranged on the anchored bars to gauge its strain values. The layout of strain gauges of the anchored bars is shown in Figure 6. Under the excitation of the ground motions, the peak strains of the anchored bars are shown in Figure 15.

Figure 15 shows that the peak strains of the anchored bars of the transfer-purge chamber scale model structure are small, and the anchored bars have sufficient anchoring strength. The peak strains of the anchored bars increase gradually with the increase in PGA. Under the earthquake conditions, the connection between the transfer-purge chamber and the nuclear fuel building is reliable without overturning. Comparing the peak strains of anchored rebars, the peak strain under excitation of MXDB is greater than that under excitation of MZQP and YJ.

#### 3.4.2. Steel Plates

**Figure 16:**
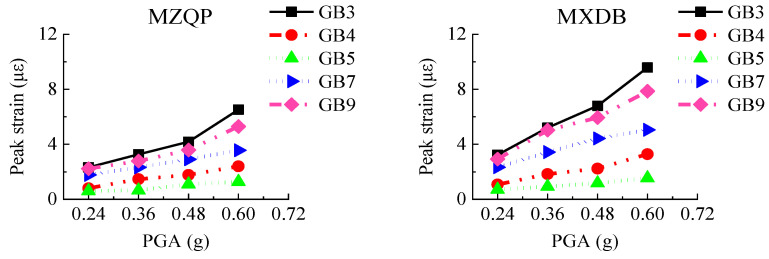

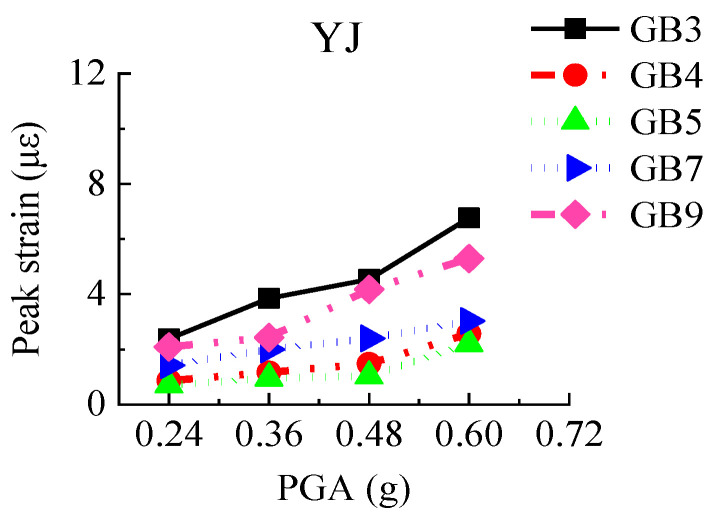
Peak strains of steel plates.

The transfer-purge chamber was constructed with an SC structure. The steel plates mainly bear the tensile stress and the shear stress as a reinforcing member in the SC structure. The layout of strain gauges of the steel plates is shown in Figure 7. Under the excitation of the ground motions, the peak strain values of the steel plates in the scale model structure are shown in Figure 16.

Figure 16 shows that the peak strains of the steel plates are generally small, and the peak strains are much smaller than the yield strain of Q235 steel (1140 με). With the increase in PGA, the peak strains of the steel plates gradually increased. The strains of rosette gages GB3, GB7, and GB8 located on the overhanging edge of the transfer chamber are significantly greater than those on other locations, which indicates that the stress at the overhanging edge in the transfer-purge chamber is higher. The peak strains of the steel plates under excitation of MXDB are slightly larger than that under excitation of MZQP and YJ. For example, when the PGA is 0.60 g, the peak strain of GB3 is 9.58 με under excitation of MXDB, 6.52 με under excitation of MZQP, and 6.76 με under excitation of YJ. These results are related to the acceleration response spectrum characteristics of MXDB, MZQP, and YJ.

#### 3.4.3. Concrete

To obtain the peak strains of the concrete of the transfer-purge chamber scale model structure, the strain gauges, named TE1–TE19, were concreted in the concrete. At the same time, three 50 mm × 150 mm openings were cut in the outer steel plate, and three strain gauges, named KC1–KC3, were mounted on the exposed concrete surface. The layout of strain gauges of the concrete is shown in Figure 8.

**Figure 17:**
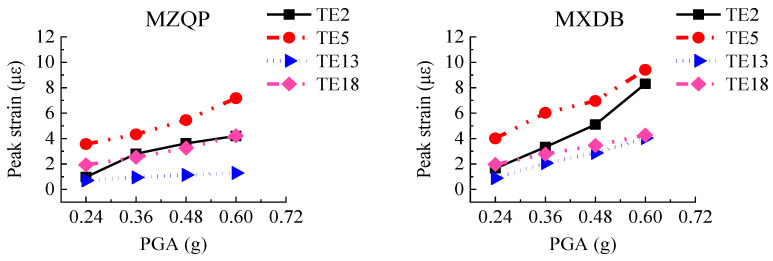

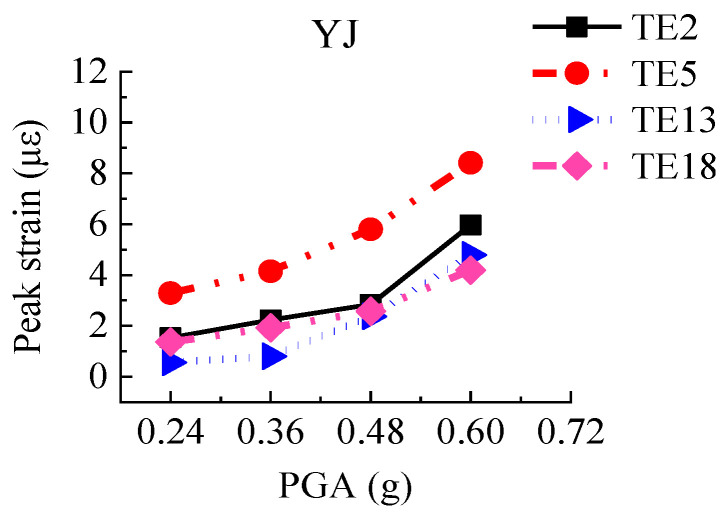
Peak strains of concrete.

For each test case, the peak strains of the concrete strain gauges are shown in Figure 17. As the PGA increases, the peak strains of the concrete gradually increase. The peak strains of the concrete are generally small and are much smaller than the compressive strain of C10 concrete (1250 με). Located on the overhanging edge of the transfer chamber, TE5 is significantly greater than TE2, TE13, and TE18, which indicates that the stress at the overhanging edge in the transfer-purge chamber is higher. The peak strains of the concrete under excitation of ground motion MXDB are slightly larger than that under excitation of ground motion MZQP and YJ. For example, when the PGA is 0.60 g, the peak strain of TE5 is 9.41 με under excitation of ground motion MXDB, 7.18 με under excitation of ground motion MZQP, and 8.38 με under excitation of ground motion YJ. It is relative to the frequency spectral characteristics of the input ground motions.

**Figure 18:**
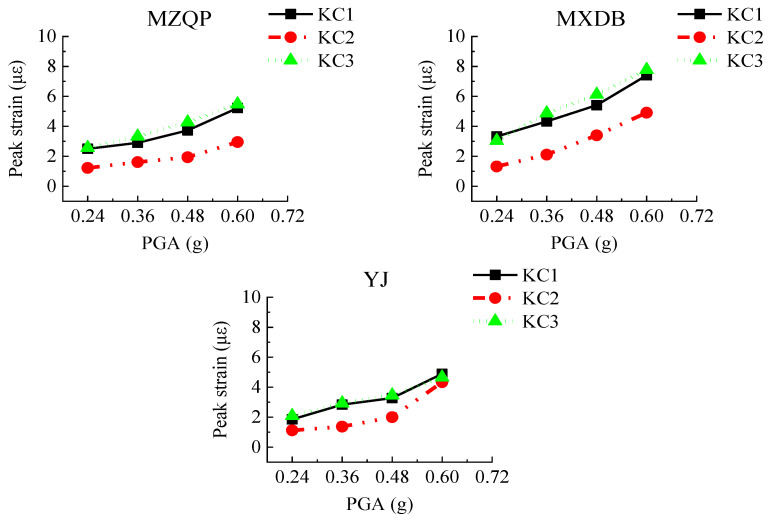
Peak strains of the exposed concrete surface.

Figure 18 shows the peak strains of KC1–KC3 in each test case. As the PGA increases, the peak strains of the concrete surface gradually increase. In Figure 18, it can be seen that the peak strains of KC1–KC3 under excitation of MZQP and YJ are relatively close and slightly smaller than those under excitation of MXDB. It is a reason that the amplitudes of the high-frequency components of MXDB are great. The maximum strain of KC1–KC3 in the transfer-purge chamber scale model structure was 7.77 με. Under the ground motions, the concrete in the transfer-purge chamber has sufficient strength to ensure the safety and reliability of the transfer-purge chamber.

## 4. Conclusions

Based on shaking table tests of the scale model structure of the transfer-purge chamber located on the top of the nuclear reactor building, the seismic performance and the dynamic characteristics of the transfer-purge chamber under earthquake loads were analyzed. The main conclusions are as follows:

(1) The maximum value of PGA in the tests was 0.60 g, and the scale model structure of the transfer-purge chamber was intact without obvious deformation or damage under the ground motions. However, there are microlesions in the joint part of the transfer-purge chamber and the base. There was no obvious crack in the concrete, the steel plates did not buckle or yield, and the welds were perfect. The steel plate is firmly attached to the concrete, and the steel plate and concrete work together well. Under seismic excitation, the overhang of the transfer-purge chamber has sufficient strength and stiffness to ensure that the structure will not overturn or be seriously damaged.
(2) Before the tests, the natural periods of the scale model of the transfer-purge chamber were 0.047 s in the X-direction and 0.050 s in the Y-direction. After the tests, the natural periods of the scale model of the transfer-purge chamber were 0.052 s in the X-direction and 0.051 s in the Y-direction. The natural frequencies of the transfer-purge chamber scale model exceeded 90% of the initial natural frequencies after the tests, which indicates that there was a slight decrease in stiffness of the transfer-purge chamber model structure and a microlesion in the connection of the transfer-purge chamber and the base.
(3) The acceleration response of the transfer-purge chamber was not violent under the earthquake. The acceleration response gradually increased with the height of the structure. The peak acceleration amplification factors of the transfer-purge chamber had nothing to do with the PGA of the input ground motions, but it was closely related to its spectral characteristics. Under the ground motion MZQP, the acceleration response of the structure was more violent than that under the ground motions MZQP and YJ.
(4) Under the excitation of large earthquakes, the peak strains of the concrete and the steel plate of the transfer-purge chamber both were small, far from reaching the yield strength of the material. From the standpoint of structural safety, the design of the transfer-purge chamber is conservative. It is suggested that the design strength of the transfer-purge chamber should be properly reduced under the condition of meeting the requirements of shielding radiation performance.

## Figures and Tables

**Table 1:** Dynamic similitude relations of model structure.

Physical Type	Physical Quantity	Similitude Relation	Similitude Ratio
Geometry property	Length *l*	$S_{l}$	1/4.5
	Displacement *d*	$S_{d}=S_{l}$	1/4.5
Material property	Elastic modulus *E*	$S_{E}$	0.32
Dynamic property	Density *ρ*	$S_{\rho }=S_{E}/\left(S_{l}S_{a}\right)$	1.2
	Acceleration *a*	$S_{a}$	1.2
	Time *t*	$S_{t}=\sqrt{S_{l}/S_{a}}$	0.43
	Frequency *f*	$S_{f}=1/S_{t}$	2.33
	Mass *m*	$S_{m}=S_{\rho }S_{l}{}^{3}$	1.32 × 10^−2^

**Table 2:** The names of sensors in the shaking table tests.

Sensors Name	Interpretation	Data Type
A	Piezoelectric accelerometer	Acceleration of the model
TE	Embedded concrete strain gauge	Concrete strain
KC	Strain gauge	Concrete strain
GB	Strain rosette	Steel plate strain
CJ	Strain gauge	Tie bars strain

**Table 3:** Interpretation of the ground motions selected in the shaking table tests.

Ground Motions	Interpretation
MZQP	Acceleration record at station MZQP from Ms7.9 Wenchuan earthquake of 12 May 2008
MXDB	Acceleration record at station MXDB from Ms7.9 Wenchuan earthquake of 12 May 2008
YJ	Artificial acceleration time-history corresponding to the acceleration response spectra at the engineering site of Yangjiang nuclear power plant in China
WN	White noise

**Table 4:** Test cases for the shaking table tests.

NO.	Test ID	Input Direction	PGA (g)
1	WN-1	X&Y&Z	0.05:0.05:0.05
2	MZQP-0.24	X&Y&Z	0.24:0.24:0.24
3	MXDB-0.24	X&Y&Z	0.24:0.24:0.24
4	YJ-0.24	X&Y&Z	0.24:0.24:0.24
5	WN-2	X&Y&Z	0.05:0.05:0.05
6	MZQP-0.36	X&Y&Z	0.36:0.36:0.36
7	MXDB-0.36	X&Y&Z	0.36:0.36:0.36
8	YJ-0.36	X&Y&Z	0.36:0.36:0.36
9	WN-3	X&Y&Z	0.05:0.05:0.05
10	MZQP-0.48	X&Y&Z	0.48:0.48:0.48
11	MXDB-0.48	X&Y&Z	0.48:0.48:0.48
12	YJ-0.48	X&Y&Z	0.48:0.48:0.48
13	WN-4	X&Y&Z	0.05:0.05:0.05
14	MZQP-0.60	X&Y&Z	0.60:0.60:0.60
15	MXDB-0.60	X&Y&Z	0.60:0.60:0.60
16	YJ-0.60	X&Y&Z	0.60:0.60:0.60
17	WN-5	X&Y&Z	0.05:0.05:0.05

**Table 5:** The predominant periods and amplitudes of the acceleration response spectra of ground motions with a PGA of 0.24 g and a damping ratio of 0.04.

Ground Motion	X-Direction		Y-Direction		Z-Direction	
	Predominant Period (s)	Amplitude of Sa (g)	Predominant Period (s)	Amplitude of Sa (g)	Predominant Period (s)	Amplitude of Sa (g)
MZQP	0.042	1.20	0.029	0.74	0.040	1.48
MXDB	0.076	1.10	0.044	1.13	0.057	1.45
YJ	0.095	0.98	0.116	1.17	0.088	1.11

**Table 6:** Natural frequencies of the transfer-purge chamber scale model.

Test Cases	X-Direction (E-W)				Y-Direction (N-S)			
	*f* (Hz)	*T* (s)	$\frac{f}{f_{ini}}\times 100\%$ (%)	$\frac{R}{R_{ini}}\times 100\%$ (%)	*f* (Hz)	*T* (s)	$\frac{f}{f_{ini}}\times 100\%$ (%)	$\frac{R}{R_{ini}}\times 100\%$ (%)
WN-1	21.30	0.047	100	100	20.20	0.050	100	100
WN-2	20.60	0.049	96.7	93.5	20.00	0.050	99.0	98.0
WN-3	19.70	0.051	92.5	85.6	19.62	0.051	97.1	94.4
WN-4	19.62	0.051	92.1	84.9	19.58	0.051	96.9	94.0
WN-5	19.40	0.052	91.1	83.0	19.58	0.051	96.9	94.0

Notes: *f* is the natural frequency of the structure; *T* is the natural period of the structure; *f_ini_* is the initial natural frequency of the structure; *R* is the stiffness of the structure; and *R_ini_* is the initial stiffness of the structure.

**Table 7:** Damping ratios of the transfer-purge chamber scale model.

Test Cases	X-Direction (E-W)		Y-Direction (N-S)	
	*ξ* (%)	$\frac{\xi_{\Delta }}{\xi_{ini}}\times 100\%$ (%)	*ξ* (%)	$\frac{\xi_{\Delta }}{\xi_{ini}}\times 100\%$ (%)
WN-1	2.3	0	2.3	0
WN-2	2.6	13	2.4	4.3
WN-3	3	30.4	2.6	13
WN-4	3.1	34.8	2.8	21.7
WN-5	3.1	34.8	2.8	21.7

Notes: *ξ* is the damping ratio of the structure; *ξ*_Δ_ is the increment of damping ratio of the structure; and *ξ_ini_* is the initial damping ratio of the structure.

## Data Availability

Not applicable.

## References

[1] Zhao Q., Astaneh-Asl A. (2004). Cyclic Behavior of Traditional and Innovative Composite Shear Walls. J. Struct. Eng..

[2] Guo L., Li R., Rong Q., Zhang S. (2012). Cyclic behavior of SPSW and CSPSW in composite frame. Thin-Walled Struct..

[3] Wright H., Gallocher S. (1995). The behaviour of composite walling under construction and service loading. J. Constr. Steel Res..

[4] Wright H. (1998). The axial load behaviour of composite walling. J. Constr. Steel Res..

[5] Akiyama H., Sekimoto H., Tanaka M., Inoue K., Fukihara M., Okuda Y. 1/10th scale model test of inner concrete structure composed of concrete filled steel bearing wall. Proceedings of the Transactions of the 10th International Conference on Structural Mechanics in Reactor Technology (SMiRT-10).

[6] Usami S., Akiyama H., Narikawa M., Hara K., Takeuchi M., Sasaki N. Study on Concrete Filled Steel Structures for Nuclear Power Plants (part 2). Compressive Loading Tests on Wall Members. Proceedings of the Transactions of the 13th International Conference on Structural Mechanics in Reactor Technology.

[7] Takeuchi M., Narikawa M., Matsuo I., Hara K., Usami S. (1998). Study on a concrete filled structure for nuclear power plants. Nucl. Eng. Des..

[8] Sasaki N., Akiyama H., Narikawa M., Hara K., Takeuchi M., Usami S. Study on a concrete filled steel structure for nuclear power plants (part 3). Shear and bending loading tests on wall member. Proceedings of the Transactions of the 13th International Conference on Structural Mechanics in Reactor Technology.

[9] Takeda T., Yamaguchi T., Nakayama T., Akiyama K., Kato Y. Experimental study on shear characteristics of a concrete filled steel plate wall. Proceedings of the Transactions of the 13th International Conference on Structural Mechanics in Reactor Technology.

[10] Ozaki M., Akita S., Osuga H., Nakayama T., Adachi N. (2004). Study on steel plate reinforced concrete panels subjected to cyclic in-plane shear. Nucl. Eng. Des..

[11] Eom T.-S., Park H.-G., Lee C.-H., Kim J.-H., Chang I.-H. (2009). Behavior of Double Skin Composite Wall Subjected to In-Plane Cyclic Loading. J. Struct. Eng..

[12] Hossain K., Wright H.D. (2004). Experimental and theoretical behavior of composite wall under in-plane shear. J. Constr. Steel Res..

[13] Hossain K.M.A., Wright H.D. (1998). Performance of Profiled Concrete Shear Panels. J. Struct. Eng..

[14] Wright H.D., Hossain K. (1997). In-plane shear behaviour of profiled steel sheeting. Thin-Walled Struct..

[15] Sener K.C., Varma A. (2014). Steel-plate composite walls: Experimental database and design for out-of-plane shear. J. Constr. Steel Res..

[16] Sener K.C., Varma A.H., Ayhan D. (2015). Steel-plate composite (SC) walls: Out-of-plane flexural behavior, database, and design. J. Constr. Steel Res..

[17] Sener K.C., Varma A.H., Malushte S.R., Coogler K. Experimental database of SC composite specimens tested under out-of-plane shear loading. Proceedings of the 22st International Conference on Structural Mechanics in Reactor Technology 2013.

[18] Zhang Y.J., Li X.J. (2015). Experimental research on seismic behavior of wall component with double steel plates and infill concrete. Eng. J. Wuhan Univ..

[19] Zhang K., Varma A.H., Malushte S.R., Gallocher S. (2014). Effect of shear connectors on local buckling and composite action in steel concrete composite walls. Nucl. Eng. Des..

[20] Li X.H., Li X.J. (2017). Steel plates and concrete filled composite shear walls related nuclear structural engineering: Experimental study for out-of-plane cyclic loading. Nucl. Eng. Des..

[21] Li X.J., Li X.H. (2017). Study on in-plane flexural behavior of double steel plates and concrete infill composite shear walls for nuclear engineering. Eng. Mech..

[22] Yang Y. (2015). Seismic Behavior of Double Steel-Concrete Composite Structures in Nuclear Engineering. Ph.D. Thesis.

[23] Xiong F., He T., Zhou N. (2015). Study on the shear strength of double steel plate composite shear wall in nuclear plant. J. Hunan Univ. (Nat. Sci.).

[24] Rogers R.C., Hermann G.E. (2019). Hindbrain astrocytes and glucose counter-regulation. Physiol. Behav..

[25] Li N., Gu J.P. (2008). Numerical analysis of transfer and purge chamber for China Experimental Fast Reactor. At. Energy Sci. Technol..

[26] Zhang M.Z. (1997). Study on similitude laws for shaking table tests. Earthq. Eng. Eng. Vib..

[27] Liu J.B., Du X.L. (2005). Structural Dynamics.

